# Supplementary material for: Sampling bias overestimates climate change impacts on forest growth in the southwestern United States
Source: Nat Commun. 2018 Dec 17;9:5336. doi: 10.1038/s41467-018-07800-y (PMC6297350; doi:10.1038/s41467-018-07800-y)
Supplement: Supplementary file 1 — Supplementary Information [file 41467_2018_7800_MOESM1_ESM.pdf]

Supplementary material to

Sampling bias overestimates climate change impacts on forest growth in the southwestern United States

S. Klesse et al.

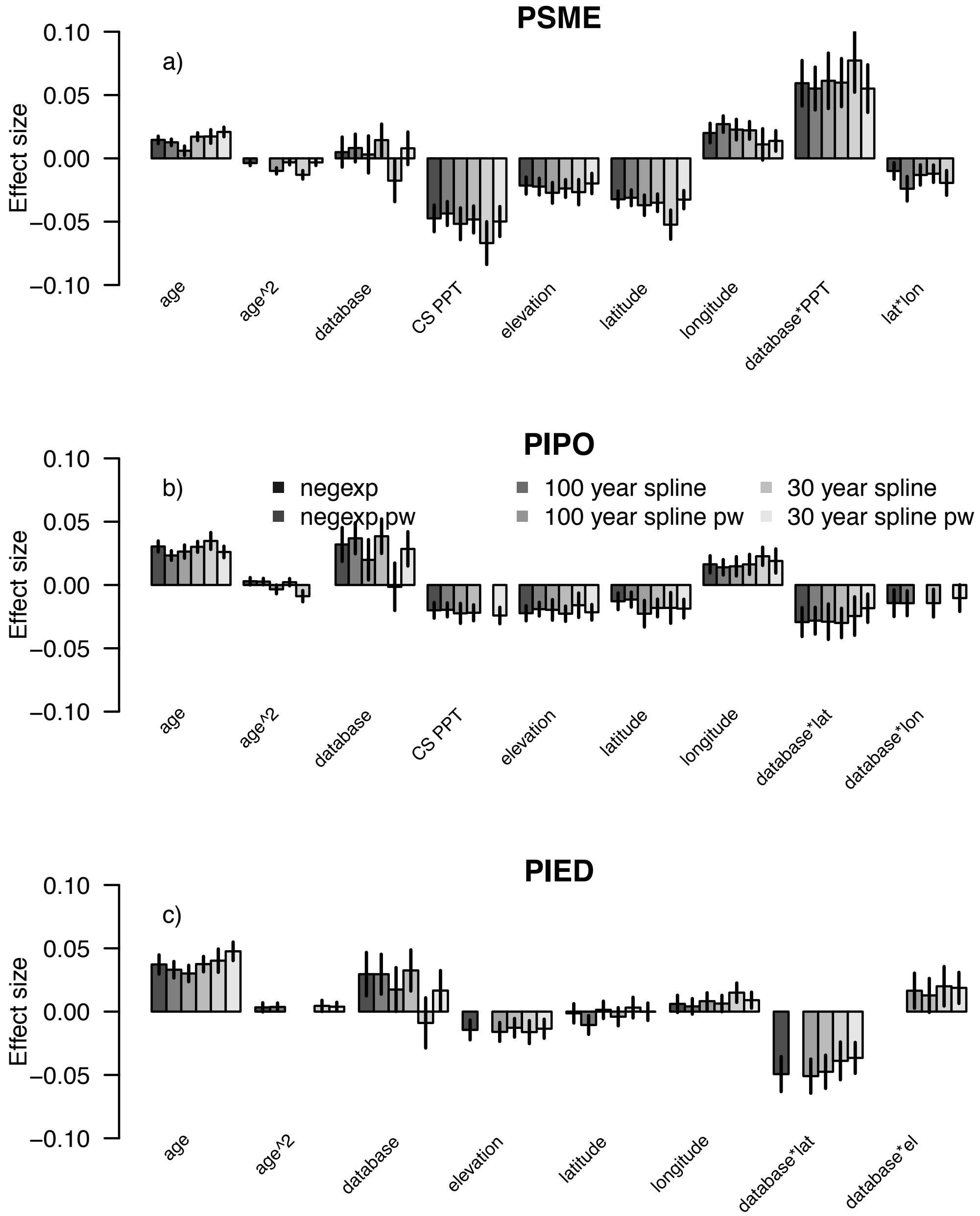

Supplementary Figure 1: Estimated effects from linear mixed-effects models explaining growth variability (SD of detrended tree-ring time series)of the three species a) Douglas-fir (PSME), b) ponderosa pine (PIPO), and c) common pinyon (PIED). The six bars from left to right are based on time-series that were: 1) 30-year spline detrended, 2) 30-year spline detrended + prewhitened (i.e. auto-correlation removed), 3) 100-year spline detrended, 4) 100-year spline detrended + prewhitened, 5) negative exponential curve detrended, 6) negative exponential curve detrended + prewhitened. Vertical lines show the 95% confidence intervals of each effect. The database parameter shows the effect of ITRDB samples compared to FIA samples (ITRDB minus FIA). CS PPT is average cool season

precipitation. Only the effects of variables are shown that ended up in at least 4 of the 6 best models per species.

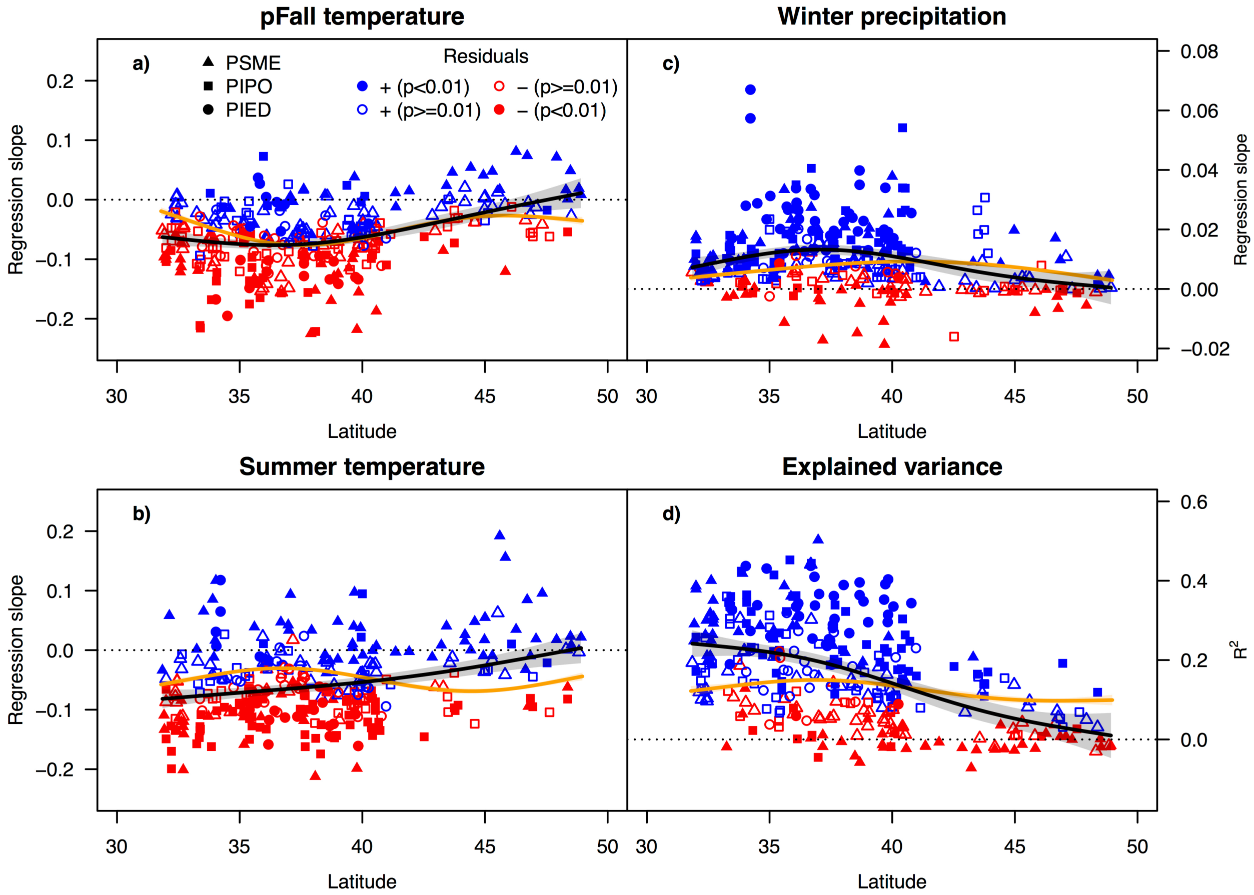

Supplementary Figure 2: Distribution of regression model statistics across latitude, including median regression slopes with respect to three climate predictors (a-c) and median explained variance (d; R^2), based on all time series per ITRDB study site. Climate predictors in the multiple regression model were: mean maximum temperature of the previous fall (August-October: a); current summer (May-July: c); and cumulative cool season precipitation (November-March: b). Filled symbols denote significant differences ($p<0.01$) between the ITRDB time series and surrounding FIA time series, assessed via 1000 iterations of bootstrapped Wilcoxon tests. The black line shows a smoothed fit through the ITRDB data (of all species), the orange line is the fit through the FIA data (data points not shown). Shading indicates the 95% confidence interval.

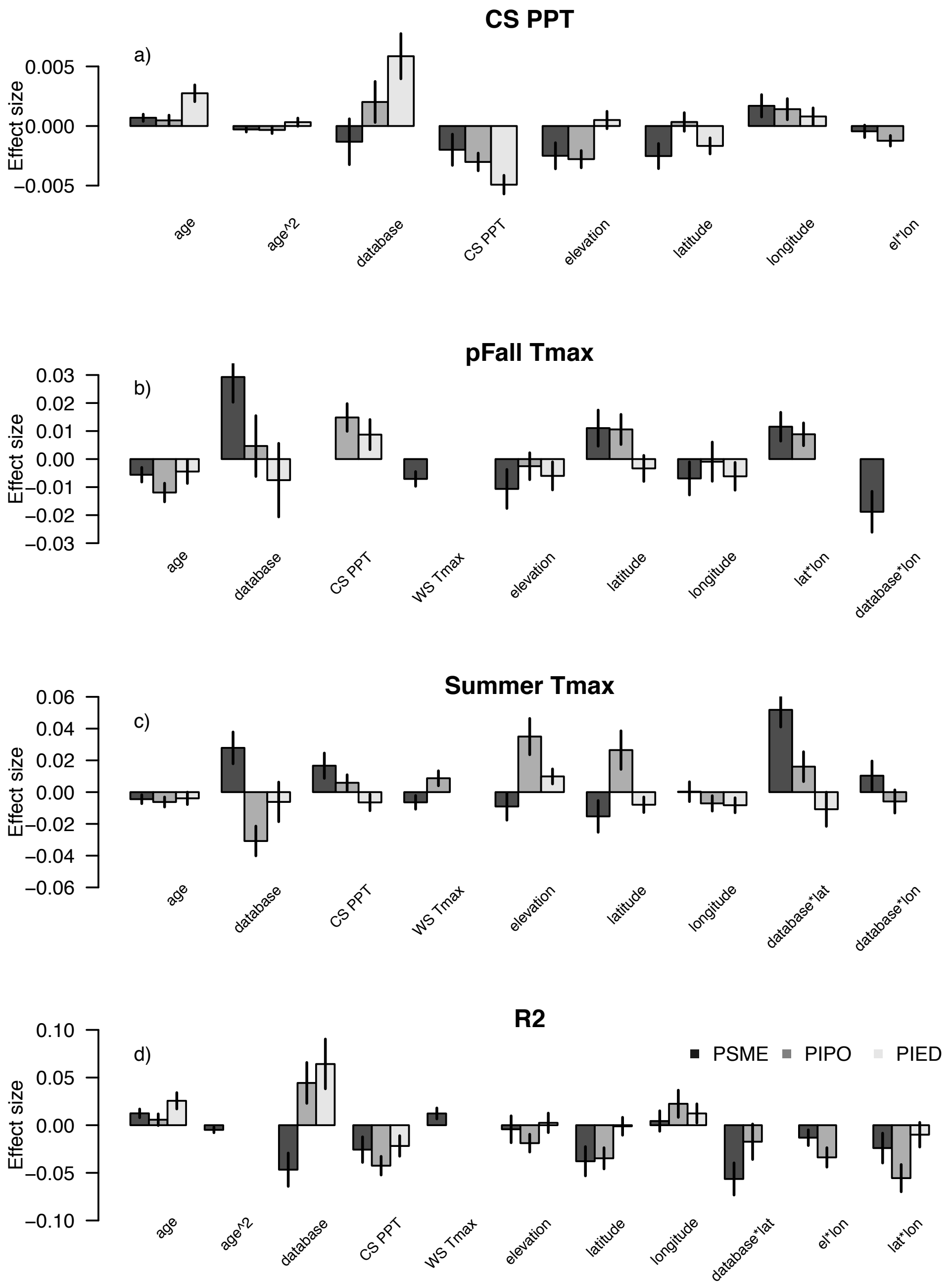

Supplementary Figure 3: Estimated effects from linear mixed-effects models explaining variation among all detrended (with a negative exponential curve) time series in climate sensitivities (the sensitivity of relative tree growth to a) cool season precipitation, CS PPT, b) mean maximum temperature of previous fall, pFall Tmax, and c) current summer, Summer Tmax) as well as d) growth variation explained by those climate variables (R^2). From left to right, the three bars indicate the effects inferred for the three study species - Douglas-fir (PSME), ponderosa pine (PIPO), and common pinyon (PIED). Vertical lines show the 95% confidence intervals of each effect. The database effect is the contrast of ITRDB minus FIA.

CS PPT is average cool season precipitation, and WS Tmax the warm season mean maximum temperature (average of previous fall and current summer).

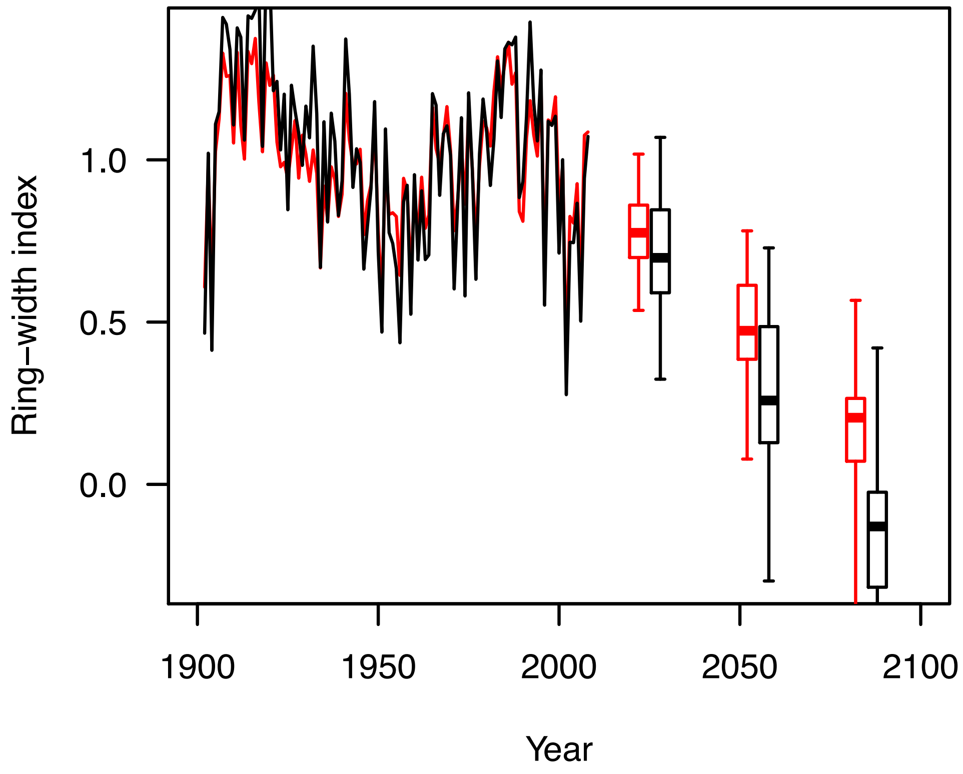

Supplementary Figure 4: Alternative projection of Regional ring-width index (RWI) chronologies for the U.S. Southwest replacing precipitation and mean maximum temperature with climatic water balance and vapor pressure deficit as predictors of tree growth based on the forest inventory sample (FIA, in red) vs. targeted sample (ITRDB, in black). Boxplots show projected mean growth for 2010-2039, 2040-2069, and 2070-2099 under the "business-as-usual" scenario RCP8.5 across 15 different Atmosphere-Ocean Global Circulation Models (GCM) from the CMIP5 ensemble. Bold horizontal lines denote the median of the projections, the boxes denote the interquartile range, and whiskers extend to the most extreme future RWI values arising from GCM-projected future climate. Instead of a relative difference between FIA and ITRDB of 29% this alternative projection results in a 30% relative difference in growth decrease.

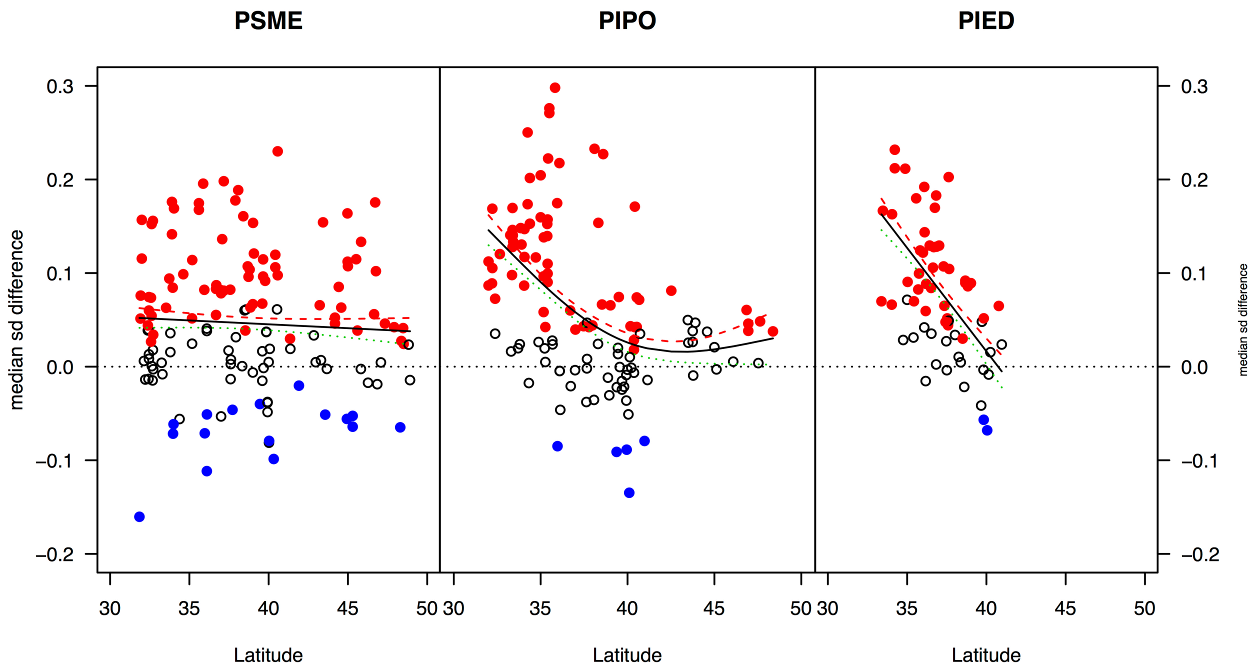

Supplementary Figure 5: Difference between the median standard deviation of ring-width index (SD(RWI)) of ITRDB timeseries compared against surrounding FIA time series, after detrending using a 30-year spline with a 50% frequency cut-off at 30 years. Contrasts are filled red if the median ITRDB SD is significantly greater than the median FIA SD ($p<0.01$, Wilcoxon test); blue if the difference is significantly negative. The smoothed regression line is the expected difference between the median SD(RWI) of the two samples with respect to latitude, with 95% confidence intervals. Compare to Figure 3, which shows the same comparison but a different method of detrending the ring-width time series.

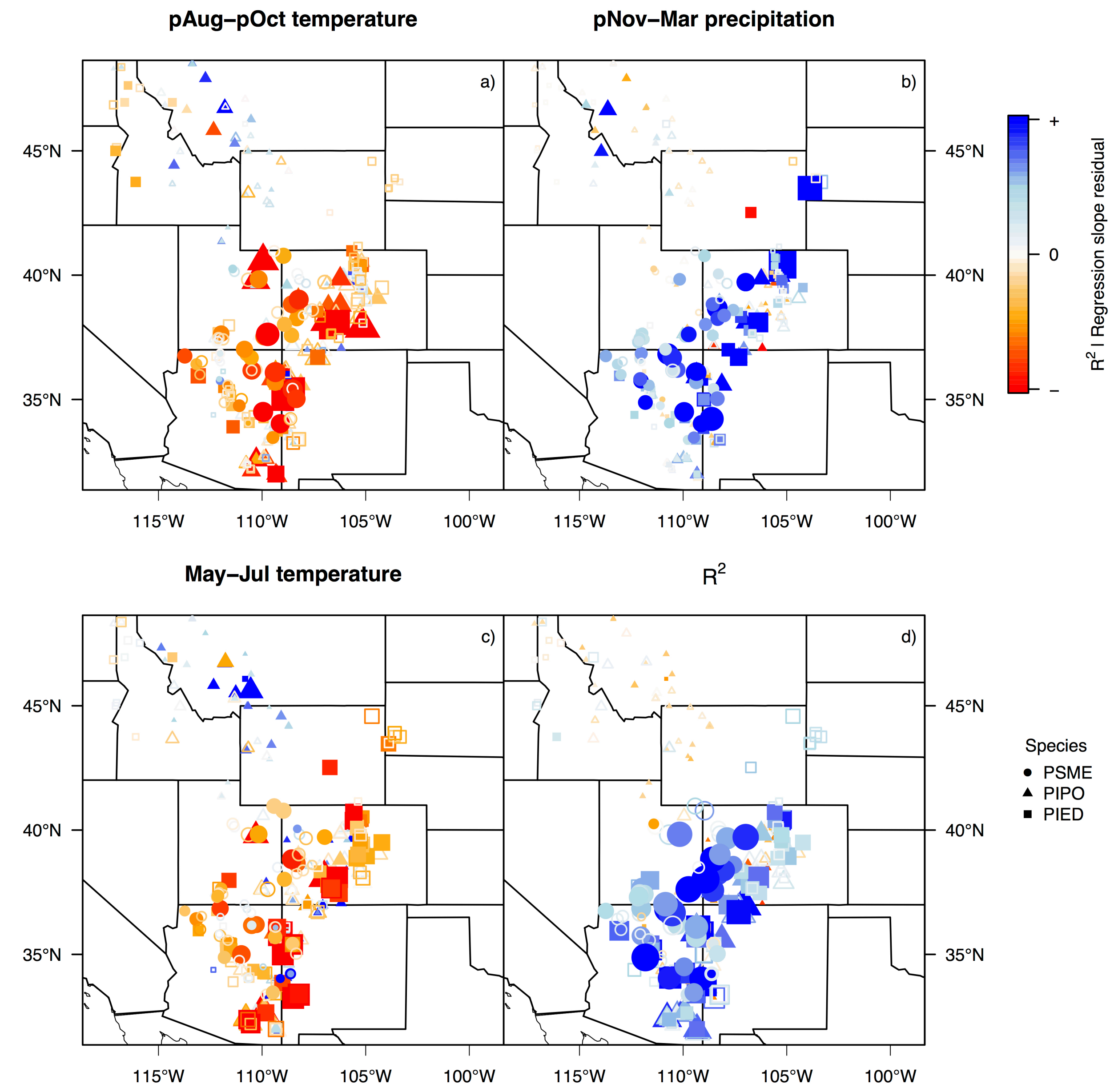

Supplementary Figure 6: Median climate sensitivity and explained variance of targeted (ITRDB) vs. forest inventory (FIA) samples, as in Fig. 4, but following the use of a 30-year spline with a 50% frequency cut-off to detrend ring-width time series. Symbol size indicates the magnitude of (a-c) sensitivity to three climate variables (regression slopes) and (d) explained variance (R^2), with positive contrasts (ITRDB minus FIA) in blue and negative contrasts in orange to red. Filled symbols denote significant differences ($p<0.01$, two-sided Wilcoxon-test) between ITRDB and surrounding FIA time series.

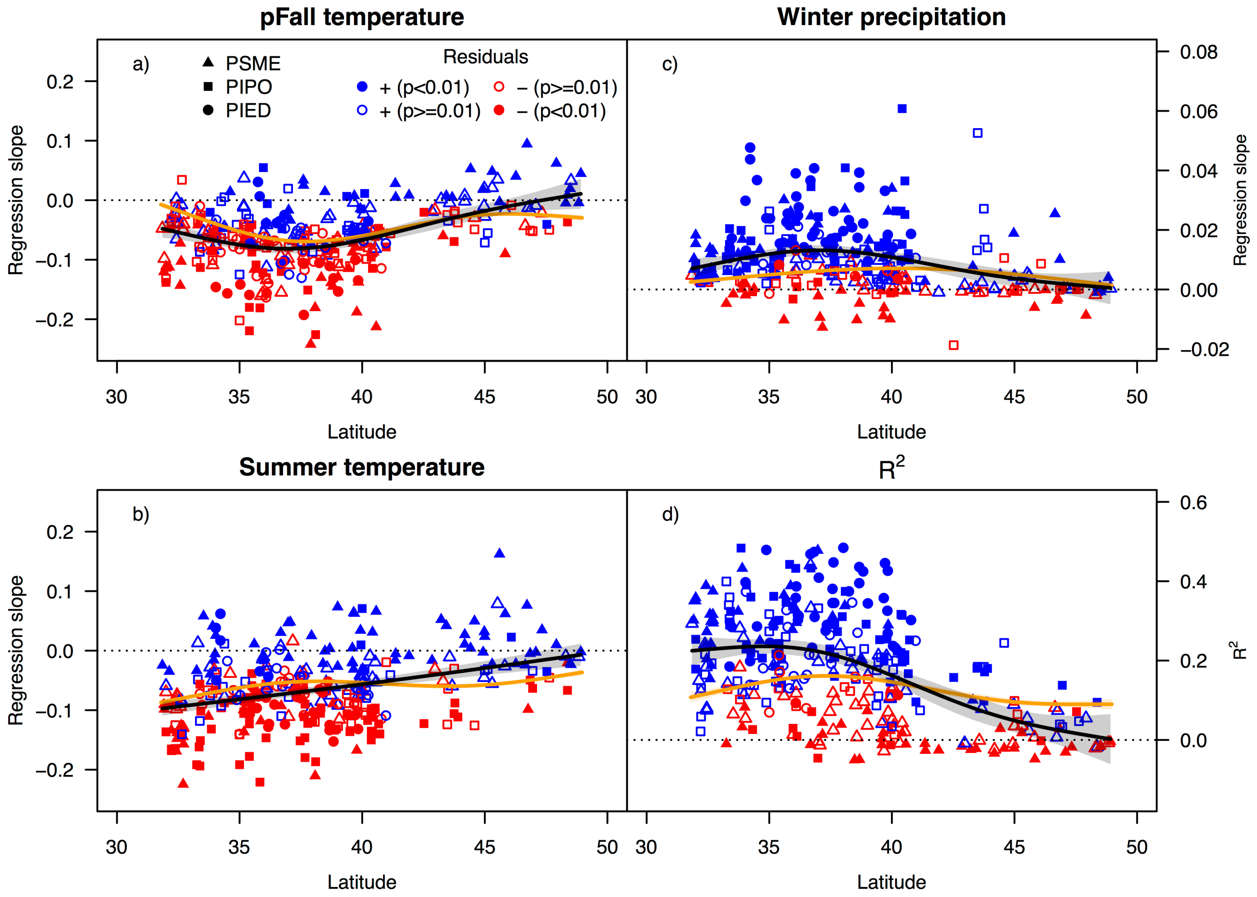

Supplementary Figure 7: Distribution of regression model statistics across latitude, including median regression slopes with respect to three climate predictors (a-c) and median explained variance (d; R^2), based on all time series per ITRDB study site as in Figure S2, but following 30-year spline detrending of the tree-ring time series. Climate predictors in the multiple regression model were: mean maximum temperature of the previous fall (August-October: a); current summer (May-July: c); and cumulative cool season precipitation (November-March: b). Filled symbols denote significant differences ($p<0.01$) between the ITRDB time series and surrounding FIA time series, assessed via 1000 iterations of bootstrapped Wilcoxon tests. The black line shows a smoothed fit through the ITRDB data (of all species), the orange line is the fit through the FIA data (data points not shown). Shadings around the regression lines show the 95% confidence interval.
