# Supplementary material for: Sampling bias overestimates climate change impacts on forest growth in the southwestern United States
Source: Nat Commun. 2018 Dec 17;9:5336. doi: 10.1038/s41467-018-07800-y (PMC6297350; doi:10.1038/s41467-018-07800-y)
Supplement: Supplementary file 3 — Reporting Summary [file 41467_2018_7800_MOESM3_ESM.pdf]

nature research

Corresponding author(s): Stefan Klesse

Reporting Summary

Nature Research wishes to improve the reproducibility of the work that we publish. This form provides structure for consistency and transparency in reporting. For further information on Nature Research policies, see Authors & Referees and the Editorial Policy Checklist.

Statistical parameters

When statistical analyses are reported, confirm that the following items are present in the relevant location (e.g. figure legend, table legend, main text, or Methods section).

n/a	Confirmed	
☐	☒	The exact sample size (*n*) for each experimental group/condition, given as a discrete number and unit of measurement
☐	☒	An indication of whether measurements were taken from distinct samples or whether the same sample was measured repeatedly
☐	☒	The statistical test(s) used AND whether they are one- or two-sided<br>*Only common tests should be described solely by name; describe more complex techniques in the Methods section.*
☐	☐	A description of all covariates tested
☒	☐	A description of any assumptions or corrections, such as tests of normality and adjustment for multiple comparisons
☐	☒	A full description of the statistics including central tendency (e.g. means) or other basic estimates (e.g. regression coefficient) AND variation (e.g. standard deviation) or associated estimates of uncertainty (e.g. confidence intervals)
☐	☒	For null hypothesis testing, the test statistic (e.g. *F*, *t*, *r*) with confidence intervals, effect sizes, degrees of freedom and *P* value noted<br>*Give P values as exact values whenever suitable.*
☒	☐	For Bayesian analysis, information on the choice of priors and Markov chain Monte Carlo settings
☐	☒	For hierarchical and complex designs, identification of the appropriate level for tests and full reporting of outcomes
☐	☒	Estimates of effect sizes (e.g. Cohen's *d*, Pearson's *r*), indicating how they were calculated
☐	☒	Clearly defined error bars<br>*State explicitly what error bars represent (e.g. SD, SE, CI)*

Our web collection on statistics for biologists may be useful.

Software and code

Policy information about availability of computer code

Data collection	Tree-ring measurements in various programs: e.g. TSAP, Tellervo, J2X<br>crossdating: COFECHA
Data analysis	Data was analysed in R Studio Version 1.1.447,<br>R version: R version 3.3.2<br>prominent R packages used: dplR, lme4, MuMIn, fossil

For manuscripts utilizing custom algorithms or software that are central to the research but not yet described in published literature, software must be made available to editors/reviewers upon request. We strongly encourage code deposition in a community repository (e.g. GitHub). See the Nature Research guidelines for submitting code & software for further information.

Data

Policy information about availability of data

All manuscripts must include a data availability statement. This statement should provide the following information, where applicable:

- Accession codes, unique identifiers, or web links for publicly available datasets
- A list of figures that have associated raw data
- A description of any restrictions on data availability

The International Tree-Ring Data Bank is located at: https://www.ncdc.noaa.gov/paleo-search/ (11/03/2017). All other data can b
e made available upon request.
Contact R.J. DeRose for the FIA, C.D. O'Connor for the Pinaleño Mountains, and C.H. Guiterman for the Navajo Nation tree-ring d
ata.

Field-specific reporting

Please select the best fit for your research. If you are not sure, read the appropriate sections before making your selection.

☐ Life sciences ☐ Behavioural & social sciences ☒ Ecological, evolutionary & environmental sciences

For a reference copy of the document with all sections, see nature.com/authors/policies/ReportingSummary-flat.pdf

Ecological, evolutionary & environmental sciences study design

All studies must disclose on these points even when the disclosure is negative.

Study description	Quantifying the bias of sampling climate-sensitive trees (as publicly available on the International Tree-Ring Data Bank (ITRDB) on the magnitude of climate change impacts on forest growth in the U.S. Interior West.
Research sample	Our analyses focus on some of the most widespread, important, and densely sampled tree species in the Interior West – Douglas-f ir (Pseudotsuga menziesii var. glauca, PSME), ponderosa pine (Pinus ponderosa, PIPO) and common pinyon (Pinus edulis, PIED). We analyze ring-width time series from U.S. forest inventory vs. targeted (ITRDB) samples.
Sampling strategy	Local scale: comparison of ITRDB data with surrounding FIA samples within 100 km (minimum 10 samples, which are a standard minimum to calculate site level characteristics)<br>Large scale: All data witihin a linear mixed effects model framework
Data collection	tree-ring time series downloaded from the International Tree-Ring Data Bank (ITRDB) as of December 2016, and 2) tree-ring time series developed as part of the Interior West Forest Inventory and Analysis (FIA) Program. The latter collection was complement ed by two additional inventory-style datasets from a) the Pinaleño Mountains in southeastern Arizona, where sampling was performed in a systematic grid of 54 0.05-ha circular plots spaced one kilometer apart, and from b) northeastern Arizona and northwestern New Mexico, where a subset of forest inventory plots on the Navajo Nation was sampled along a climate gradient.
Timing and spatial scale	data collected in Decemeber 2016.<br>Time scale of analysis: 1930-1995 (period with maximum replication of the FIA dataset) and 1902:2008 (climate data common period)<br>Spatial scale: US Interior West
Data exclusions	At the local scale analysis we discarded those ITRDB locations with less than 10 FIA samples of the same species within a radiu s of 100 km. 10 samples are usually a minimum to build a local site chronology.
Reproducibility	all attempts to repeat the analysis were successful
Randomization	Not relevant, data analyzed on comparison of local scale (ITRDB) chronologies with surrounding forest inventory samples, and accompanied by a linear mixed effects model using latitude and longitude (among others) as explaining factors.
Blinding	blinding is not relevant in this discipline.

Did the study involve field work? ☐ Yes ☒ No

Reporting for specific materials, systems and methods

Materials & experimental systems

n/a	Involved in the study	
☒	☐	Unique biological materials
☒	☐	Antibodies
☒	☐	Eukaryotic cell lines
☒	☐	Palaeontology
☒	☐	Animals and other organisms
☒	☐	Human research participants

Methods

n/a	Involved in the study	
☒	☐	ChIP-seq
☒	☐	Flow cytometry
☒	☐	MRI-based neuroimaging
